# Effectiveness of Curcumin on Oxidative Stress in Goat Semen: Explorations Regarding Semen Quality, Sperm Apoptosis, Ultrastructure, and Markers of Oxidative Stress

**DOI:** 10.3390/antiox14101242

**Published:** 2025-10-16

**Authors:** Zhaoxiang An, Wenjuan Xun, Hanlin Zhou, Guanyu Hou, Liguang Shi

**Affiliations:** 1 Tropical Crops Genetic Resources Institute, Chinese Academy of Tropical Agricultural Sciences, Haikou 571100, China; anzx1111@163.com (Z.A.);; 2 School of Tropical Agriculture and Forestry, Hainan University, Haikou 570228, China; 3 Zhanjiang Experimental Station, Chinese Academy of Tropical Agricultural Sciences, Zhanjiang 524000, China

**Keywords:** curcuma longa, antioxidant, transmission electron microscopy, lipidomics and metabolomics, apoptotic genes

## Abstract

Oxidative stress induces reactive oxygen species (ROS) accumulation, which compromises sperm DNA integrity, cellular homeostasis, and semen quality in Hainan black goats. This study aimed to mitigate ROS-mediated sperm damage by examining the protective effects of curcumin on metabolic regulation and sperm structural integrity. Semen samples were treated in vitro with varying concentrations of curcumin (5, 25, 50 μmol/L) under oxidative stress conditions. The intermediate concentration (25 μmol/L) was most effective at enhancing sperm quality. Following treatment, sperm motility, membrane integrity, and acrosome stability were significantly improved (*p* < 0.05), while ROS levels and apoptosis rates decreased. Antioxidant enzyme activities—glutathione peroxidase (GPX, *p* < 0.05), catalase (CAT, *p* < 0.05), and superoxide dismutase (SOD, *p* < 0.05)—were markedly elevated. Metabolomic analysis identified 48 differential metabolites (*p* < 0.05), including gluconic acid, 3-hydroxybutyric acid, and argininosuccinic acid, which were mainly involved in antioxidant defense, energy metabolism (e.g., the citrate cycle), and osmoregulatory pathways. Lipidomics revealed reduced lipid peroxidation and increased polyunsaturated fatty acid content, correlating with enhanced membrane stability. Transmission and scanning electron microscopy revealed preservation of sperm ultrastructure, with reduced mitochondrial and chromatin damage. Quantitative PCR further indicated curcumin-mediated downregulation of pro-apoptotic genes (*BAX*, *Caspase3*, and *FAS*) and upregulation of the anti-apoptotic gene *BCL2* (*p* < 0.05). These findings demonstrate that Curcumin at 25 μM mitigated menadione-induced oxidative stress in goat sperm in vitro, improving antioxidant status, mitochondrial function and membrane integrity while reducing apoptosis. Multi-omic profiling supported redox and lipid homeostasis restoration. These findings establish proof-of-principle in an acute oxidative model.

## 1. Introduction

There is increasing interest in understanding the role of natural antioxidants in preserving sperm quality under oxidative stress. Spermatozoa, rich in polyunsaturated fatty acids (PUFAs) and inherently deficient in antioxidant defenses, are highly susceptible to oxidative damage [1,2,3]. Oxidative stress, defined as an imbalance between reactive oxygen species (ROS) production and antioxidant capacity, has been linked to male infertility in both humans and livestock [4,5]. In livestock, this phenomenon compromises fertility, impedes breeding programs, and threatens agricultural sustainability. Environmental pollutants, nutritional deficiencies, and physiological stressors further exacerbate oxidative stress in breeding males, leading to reduced sperm viability, motility, and fertilization capacity [6].

ROS-mediated oxidative stress induces DNA damage and apoptosis in spermatozoa [7]. Apoptosis, characterized by caspase activation, mitochondrial membrane potential disruption, phosphatidylserine externalization, and DNA fragmentation, has a direct negative impact on sperm function [8,9,10]. Apoptosis directly affects the quality of sperm and exhibits typical markers of apoptosis [11]. However, the apoptotic response of goat sperm under oxidative stress remains underexplored, limiting species-specific antioxidant interventions.

Curcumin, a polyphenolic compound derived from Curcuma longa, possesses strong antioxidant properties by scavenging ROS, inhibiting lipid peroxidation, and modulating antioxidant enzyme systems [12,13,14,15]. Its high bioavailability and favorable safety profile make it an attractive candidate for reproductive health applications [16]. Curcumin’s ability to regulate cellular pathways involved in oxidative defense, inflammation, and apoptosis further supports its potential as a therapeutic agent [17]. In addition, curcumin has been shown to enhance mitochondrial activity and preserve membrane integrity, thus protecting spermatozoa from oxidative damage such as lipid peroxidation and DNA fragmentation [18,19].

Despite these promising attributes, the specific biochemical pathways through which curcumin modulates sperm metabolism and lipid profiles remain unclear, particularly in genetically distinct breeds such as the Hainan Black goat. Native to the subtropical Hainan region of China, these goats exhibit unique physiological adaptations that may influence their response to oxidative stress and antioxidant treatment [20]. Investigating the effect of curcumin in this breed may yield valuable insights into its utility for improving sperm quality and reproductive performance in livestock.

Metabolomics, lipidomics, and proteomics represent comprehensive approaches for identifying biomarkers associated with reproductive dysfunction, offering detailed insights into molecular alterations in sperm cells [21,22]. Among these, metabolomics and lipidomics provide high-resolution phenotypic snapshots by capturing biochemical activity, making them particularly valuable for studying cellular responses to environmental stressors and pathological conditions [23,24]. These methods may offer a thorough understanding of curcumin’s impact on cellular metabolism, energy dynamics, and membrane structure.

This study aimed to evaluate the effects of curcumin supplementation on semen quality, apoptosis, ultrastructure, and oxidative stress markers in sperm exposed to oxidative stress. By applying metabolomic and lipidomic profiling, we sought to elucidate the underlying biochemical pathways affected by curcumin and to identify metabolites and lipid species that may serve as biomarkers of sperm resilience. Through this integrated approach, we provide a detailed investigation into the molecular mechanisms by which curcumin enhances sperm function and counteracts oxidative damage. Our findings support curcumin’s potential as a natural antioxidant for improving sperm quality and offer critical insights into breed-specific antioxidant strategies for sustainable livestock breeding and genetic improvement.

## 2. Materials and Methods

### 2.1. Animals

Hainan Black male goats were sourced from the Genetic Resource Preservation Farm of Hainan Province (Danzhou, Hainan, China). All procedures complied with the Standards for the Protection and Utilization of Laboratory Animals of the People’s Republic of China and were approved by the Animal Care and Use Committee of Hainan University (protocol HNUAUCC-2022-000140). Bucks were clinically healthy and exhibited normal libido.

### 2.2. Collection and Dilution of Seminal Fluid

Twenty sexually mature Hainan Black goats (*Capra hircus*), aged 1.5–2 years and weighing 35.5 ± 3.2 kg, in optimal reproductive condition were selected from a standardized breeding facility. All bucks exhibited normal libido. Semen was collected using a standardized electroejaculation protocol, starting at 3 V with 5 s pulses, followed by 0.5 V increments every 30 s up to a maximum of 6 V. Collections were performed three times per week for three consecutive weeks to generate biological replicates (*n* = 5 per group). Fresh ejaculates were retained for further analysis if they met the following criteria: volume ≥ 0.75 mL, opalescent white coloration, absence of contamination, progressive motility ≥ 80%, sperm concentration ≥ 1.5 × 10^9^ cells/mL, and normal morphology ≥ 85%.

### 2.3. Preparation of Diluent and Experimental Design

All chemicals, including D-glucose (CAS 50-99-7), D-fructose (CAS 57-48-7), Tris base (CAS 77-86-1), streptomycin sulfate (CAS 3810-74-0), dimethyl sulfoxide (DMSO, CAS 67-68-5), and curcumin (≥94% purity, CAS 458-37-7), were obtained from Sorbolol (Beijing, China). Menadione (crystalline, ≥99%, CAS 58-27-5) was purchased from Sigma-Aldrich (St. Louis, MO, USA).

The base extender was formulated as a citrate-Tris buffer containing Tris-hydroxymethyl aminomethane (27 g/L), sodium citrate (13.75 g/L), fructose (10 g/L), and streptomycin sulfate (1 × 10^6^ IU/L). Curcumin stock solutions (10 mmol/L) were prepared in DMSO and diluted to final concentrations of 5, 25, and 50 μmol/L. Menadione working solution (10 mM) was freshly prepared in DMSO and diluted to 0.1 mM [25,26].

Pooled semen samples (*n* = 5 biological replicates) were randomly assigned to five groups through stratified randomization: (1) Control: citrate-Tris buffer + 0.1% DMSO; (2) Menadione (Men): 0.1 mM menadione; (3) L-Men-Cur: 0.1 mM menadione + 5 μmol/L curcumin; (4) M-Men-Cur: 0.1 mM menadione + 25 μmol/L curcumin; (5) H-Men-Cur: 0.1 mM menadione + 50 μmol/L curcumin. All treatments maintained a final DMSO concentration of 0.1% (*v*/*v*). After additive administration, samples were equilibrated in a 37 °C water bath (Memmert WB14, Schwabach, Germany) for 10 min, followed by a 60 min incubation under atmospheric conditions. All samples were immediately stored at −80 °C following treatment preparation for subsequent omics analyses.

### 2.4. Detection of Antioxidant Properties

Total antioxidant capacity (T-AOC), superoxide dismutase (SOD), glutathione peroxidase (GSH-Px), catalase (CAT), and malondialdehyde (MDA) levels were assessed using commercial kits (A015-2-1, A001-2-2, A005-1-1, A007-1-1; Nanjing Jiancheng Bioengineering Institute, Nanjing, China), according to the manufacturer’s instructions. Absorbance was measured at 593 nm, 550 nm, 412 nm, 405 nm, and 532 nm, respectively. Reactive oxygen species (ROS) were detected using the E004-1 kit (Nanjing Jiancheng). Sperm smears were fixed in 4% paraformaldehyde in PBS for 30 min and permeabilized with 1% Triton X-100/PBS for 2 h. Samples were stained with 0.1 mM DCFH-DA for 60 min and counterstained with DAPI (Roche Diagnostics, Basel, Switzerland). Fluorescence images were captured using a laser confocal microscope (LSM 900 META, Zeiss, Oberkochen, Germany).

### 2.5. Properties Mitochondrial Function Assays

Intracellular ATP levels were determined using an ATP assay kit (A095-1-1, Nanjing Jiancheng Bioengineering Institute, Nanjing, China), following a modified protocol. Sperm suspensions (1 × 10^7^ cells/mL) were centrifuged at 600× *g* for 10 min at 4 °C. Pellets were lysed with 100 μL of extraction buffer (50 mM Tris-HCl, 1 mM EDTA, 0.1% Triton X-100, pH 7.8) and reacted with a luciferase-based reagent. Luminescence was read at 636 nm using a SpectraMax i3x multimode plate reader.

Mitochondrial membrane potential (ΔΨm) was assessed using a JC-1 staining kit (C2006, Nanjing Jiancheng Bioengineering Institute, Nanjing, China). After staining and washing, fluorescence was measured at excitation/emission wavelengths of 525/590 nm (aggregates) and 488/525 nm (monomers).

### 2.6. Metabolite Extraction and Analysis

Samples were divided into three groups: Control, Men, and Men-Cur (25 μmol/L curcumin). After thawing on ice, 100 μL aliquots were mixed with 400 μL of methanol:acetonitrile (1:1 *v*/*v*) containing 1% isotope-labeled internal standards. The mixture was vortexed and ultrasonicated for 10 min in an ice bath, followed by protein precipitation at –40 °C for 1 h. Supernatants were collected after centrifugation (12,000× *g*, 15 min, 4 °C) and subjected to UPLC-MS/MS analysis (ACQUITY UPLC, Waters Corporation (Milford, MA, USA); Xevo TQ-S, Waters).

Data were processed using ProteoWizard and XCMS for peak detection and alignment. Multivariate analysis (OPLS-DA) and univariate t-tests were conducted. Metabolites with VIP > 1, *p* < 0.05, and FC > 1.5 or <0.67 were considered significant. Pathway enrichment was performed using KEGG.

### 2.7. Lipid Extraction and LC-MS Analysis

Lipid extraction involved sequential addition of water (200 μL), MTBE (800 μL), and methanol (240 μL), followed by vortexing, ultrasonication (20 min), and centrifugation (14,000× *g*, 10 °C, 15 min). The organic phase was dried under nitrogen, reconstituted in isopropanol/acetonitrile (90:10), centrifuged, and analyzed.

UHPLC separation was performed on a Nexera LC-30A system (Shimadzu, Kyoto, Japan) with a C18 column at 45 °C and 300 μL/min flow rate. MS detection was conducted on a QExactive™ (Thermo Scientific, Waltham, MA, USA) in positive ion mode. Raw data were analyzed using LipidSearch. Differential lipids were screened by VIP > 1, FC > 1.5 or <0.67, and *p* < 0.05. Pearson correlation analysis was applied to evaluate lipid associations.

### 2.8. Sperm Ultrastructure

Sperm pellets were fixed in 2.5% glutaraldehyde, followed by 1% osmium tetroxide. Samples were dehydrated through graded ethanol, transitioned with isoamyl acetate, and dried using a critical point dryer (Quorum K850, East Sussex, UK). After gold sputtering, samples were observed under a scanning electron microscope (Hitachi SU8100, Tokyo, Japan).

For TEM, sperm were fixed in 2% glutaraldehyde, post-fixed in 1% osmium tetroxide, dehydrated, embedded in epoxy resin, and sectioned. Ultrathin sections were stained and visualized using a JEM-1400 PLUS (JEOL Ltd., Tokyo, Japan) at 80 kV.

### 2.9. Evaluation of Apoptosis

Apoptotic sperm were identified using the TUNEL assay (Click-iT™ Plus, Thermo Fisher). Samples were fixed in 4% paraformaldehyde, permeabilized with 0.1% Triton X-100, and incubated with TUNEL reaction mixture at 37 °C for 60 min. Nuclei were counterstained with DAPI and visualized under a Zeiss LSM 880 confocal microscope.

### 2.10. Quantitative Real-Time PCR

Total RNA was extracted using TRIzol (Beyotime, Shanghai, China), and cDNA was synthesized with M-MLV reverse transcriptase. Gene expression was quantified using SYBR Green-based qPCR (CFX96, Bio-Rad, Hercules, CA, USA). Relative expression levels were calculated via the 2^−ΔΔCt^ method, normalized to β-actin. Primer sequences are listed in Table 1.

### 2.11. Statistical Analyses

All data are presented as mean ± SEM. Statistical analyses were performed using SPSS 20.0 (IBM, USA). One-way ANOVA followed by LSD post hoc tests was used to assess differences among groups. Significance was defined at *p* < 0.05. Graphs were generated using GraphPad Prism 10 (GraphPad Software, San Diego, CA, USA).

## 3. Results

### 3.1. The Specific Effects of Curcumin on the Antioxidant and Metabolic Capacity of Sperm Under Oxidative Stress

**Figure 1:**
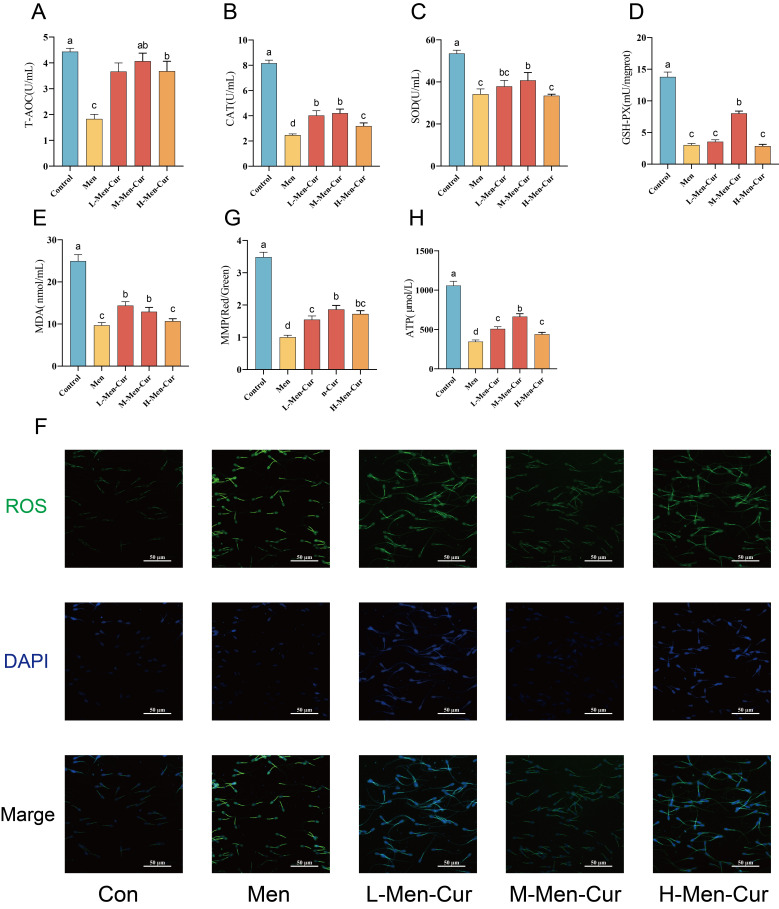
Antioxidant status of sperm after oxidative stress and modulation by curcumin. (**A**) Total antioxidant capacity (T-AOC). (**B**) Catalase (CAT) activity. (**C**) Superoxide dismutase (SOD) activity. (**D**) Glutathione peroxidase (GSH-Px) content. (**E**) Malondialdehyde (MDA) level. (**F**) Reactive oxygen species (ROS) level. (**G**) Mitochondrial membrane potential. (**H**) ATP concentration. Notes: 1. Con: citrate-Tris buffer + 0.1% DMSO; Men: 0.1 mM menadione; L-Men-Cur: 0.1 mM menadione + 5 μmol/L curcumin; M-Men-Cur: 0.1 mM menadione + 25 μmol/L curcumin; H-Men-Cur: 0.1 mM menadione + 50 μmol/L curcumin (*n* = 5 biological replicates). 2. Data are presented as mean ± SD. Groups not sharing a letter differ significantly (*p* < 0.05).

As illustrated in Figure 1, oxidative stress significantly reduced sperm T-AOC (Figure 1A), which was effectively restored by curcumin treatment. Similarly, oxidative stress markedly suppressed the activity of key antioxidant enzymes—including CAT (Figure 1B), SOD (Figure 1C) and GSH-Px (Figure 1D)—while curcumin significantly enhanced their activities. Lipid peroxidation, assessed by MDA levels (Figure 1E), increased under oxidative conditions but was significantly attenuated by curcumin. ROS accumulation, a hallmark of oxidative stress, was also significantly inhibited following curcumin treatment (Figure 1F). Furthermore, curcumin reversed oxidative stress-induced mitochondrial dysfunction by restoring mitochondrial membrane potential and ATP production, thereby supporting cellular energy homeostasis (Figure 1G,H). These findings suggest that curcumin effectively mitigates oxidative damage by enhancing both antioxidant defenses and mitochondrial function. Among the tested concentrations, the M-Men-Cur group (25 μmol/L) exhibited the most pronounced protective effect.

### 3.2. Multivariate Statistical Analysis and Clustering Analysis of the Metabolome

**Figure 2:**
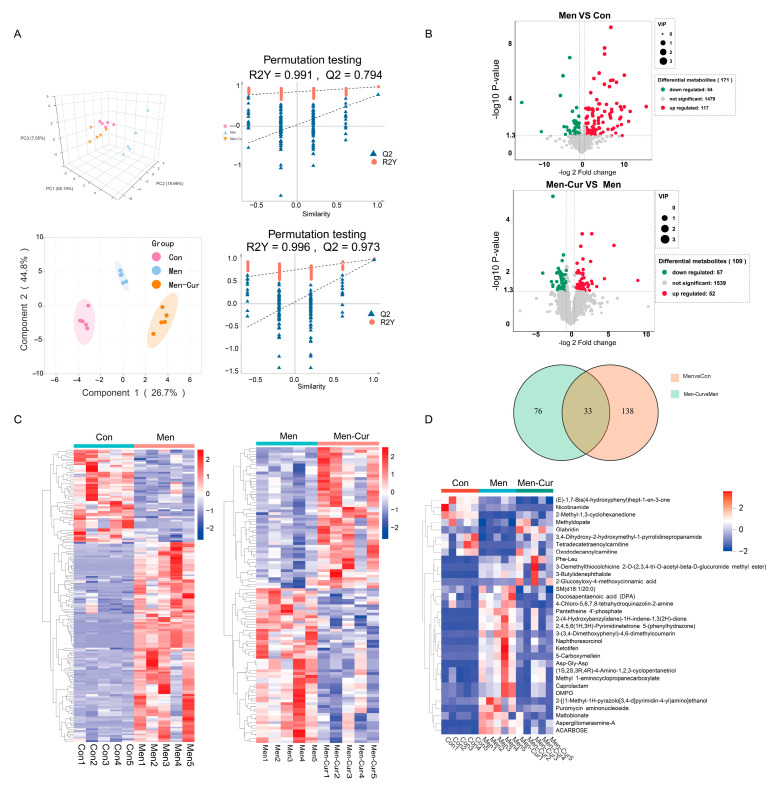
Metabolomics multivariate statistical analysis. (**A**) Principal component analysis (PCA) 3D score plot, orthogonal partial least squares–discriminant analysis (OPLS-DA) score plot, and response permutation test used to assess model validity (lower permuted R^2^/Q^2^ vs. the original model indicates reduced risk of overfitting). (**B**) Volcano plot from LC–MS comparing the two groups. The *x*-axis shows log_2_ fold change (log_2_FC) and the *y*-axis shows −log_10_(*p* value). Metabolites with log_2_FC > 0 and *p* < 0.05 are highlighted in red (up-regulated), those with log_2_FC < 0 and *p* < 0.05 in green (down-regulated), and non-significant features in gray (−log_10_ *p* < 1.3). (**C**) Heatmap of differentially abundant metabolites; colors encode relative abundance (red, higher; blue, lower). (**D**) Venn diagram showing the overlap of differential metabolites between the two groups and a heatmap of the shared metabolites (color scale as in panel C). Note: 1. Con: citrate-Tris buffer + 0.1% DMSO; Men: 0.1 mM menadione; Men-Cur: 0.1 mM menadione + 25 μmol/L curcumin (*n* = 5 biological replicates).

Multivariate analysis revealed distinct metabolic profiles in sperm subjected to oxidative stress and curcumin treatment. Principal component analysis (PCA) indicated high intra-group consistency, while orthogonal partial least squares discriminant analysis (OPLS-DA) confirmed significant separation between groups (Figure 2A), with robust model performance (Q^2^ > 0.5, R^2^Y > 0.9). Oxidative stress induced notable metabolic alterations, which were partially reversed by curcumin. Differential abundance analysis identified 171 altered metabolites in the oxidative stress group (117 upregulated, 54 downregulated) and 109 in the curcumin-treated group (52 upregulated, 57 downregulated) (Figure 2B). Hierarchical clustering demonstrated clear metabolic divergence between groups (Figure 2C). Cross-comparison revealed 33 overlapping differentially abundant metabolites (DAMs) exhibiting opposing expression patterns: those elevated under oxidative stress were downregulated following curcumin treatment, and those suppressed by stress were upregulated by curcumin (Figure 2D). These findings suggest that curcumin exerts targeted regulatory effects on metabolic alterations induced by oxidative stress.

### 3.3. Identification of Distinct Metabolites

**Figure 3:**
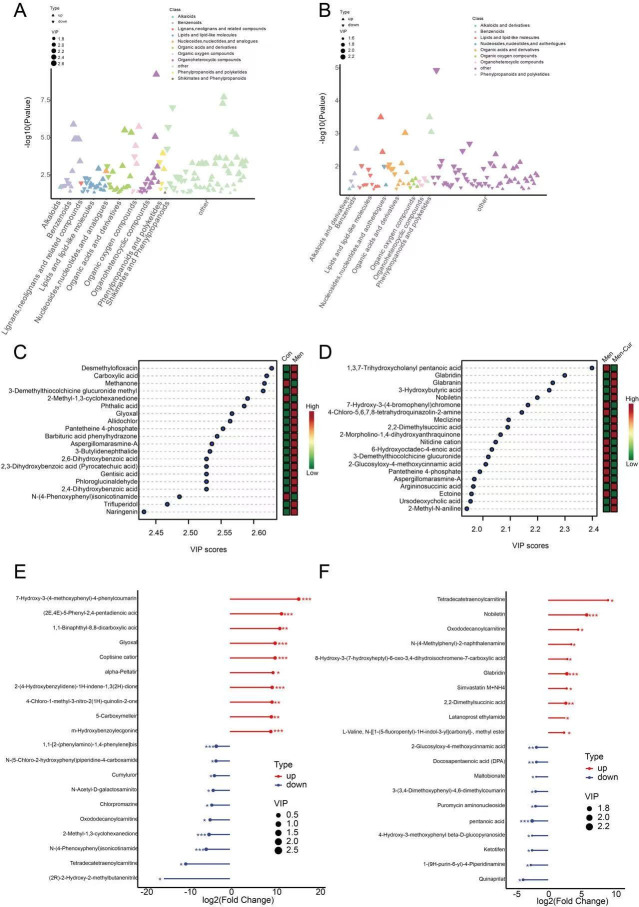
Differential metabolite screening. (**A**,**B**) Class distribution of differential metabolites in each group and the direction of regulation (up- vs. down-regulated). (**C**,**D**) OPLS-DA variable importance in projection (VIP) rank plots. Metabolites with higher VIP values contribute more to group separation; the side color scale indicates expression changes (red, up-regulated; green, down-regulated; relative to the comparator). (**E**,**F**) Log_2_ fold-change (log_2_FC) plots of differential metabolites in each group (positive values indicate up-regulation; negative values indicate down-regulation). Note: (1) Con: citrate-Tris buffer + 0.1% DMSO; (2) Men: 0.1 mM menadione; (3) Men-Cur: 0.1 mM menadione + 25 μmol/L curcumin. *, *p* value < 0.05; **, *p* value < 0.01; ***, *p* value < 0.001.

Metabolite categorization revealed that lipids and lipid-like molecules, along with organic acids and derivatives, were the predominant classes of differentially abundant metabolites in both the oxidative stress and curcumin-treated groups (Figure 3A,B). The top 20 metabolites, ranked by VIP scores, demonstrated strong discriminatory power between groups (Figure 3C,D). These metabolites were further prioritized based on their log_2_(fold change) (log_2_FC) values to characterize their expression patterns (Figure 3E,F). Notably, chlorpromazine—a compound associated with pro-oxidative effects—was elevated under oxidative stress conditions. In contrast, metabolites such as nobiletin, glabridin, docosapentaenoic acid, and ketotifen, known for their antioxidant properties, were significantly upregulated following curcumin treatment.

### 3.4. Analysis of Differential Correlations Among Metabolites

**Figure 4:**
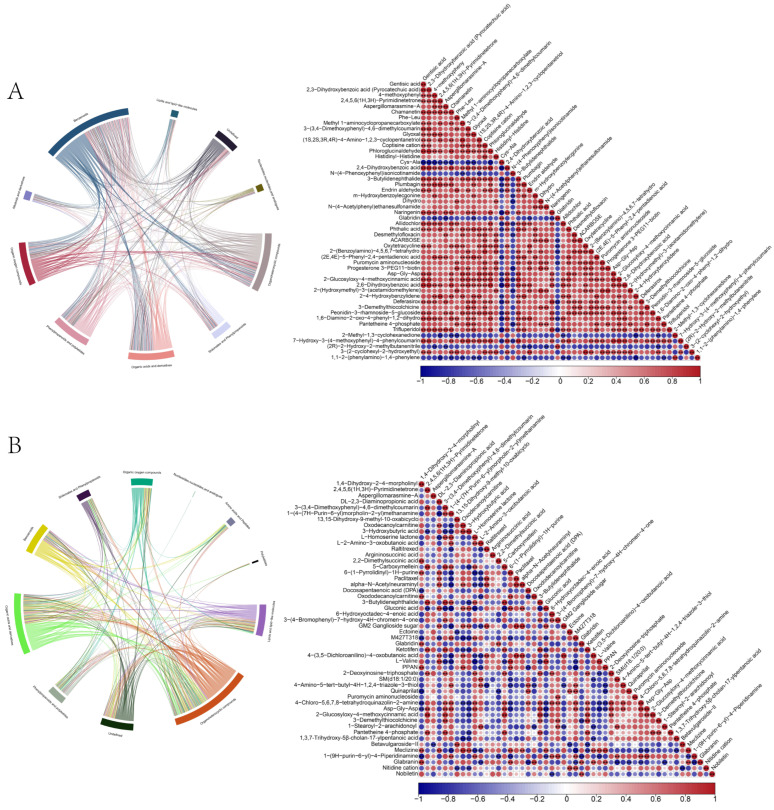
Differential metabolite correlation analysis. (**A**) Pairwise correlation heatmap of differential metabolites in the oxidative-stress group (top 50 by differential signal). (**B**) In the curcumin–treated group, class-level correlation matrix and heatmap of the top 50 differential metabolites. Cells show correlation coefficients among normalized metabolite abundances; red indicates positive correlation and blue negative correlation. Color saturation scales with the absolute correlation (stronger color = larger |r|). Metabolites are ordered by hierarchical clustering to highlight correlation structure. The correlation metric and significance criteria (e.g., Pearson or Spearman; *p*-value thresholds) follow the Methods. Note: **, *p* value < 0.01.

Spearman correlation analysis was conducted on the top 50 metabolites with the highest VIP scores. Additionally, direct correlation analysis was performed to compare metabolite relationships between the oxidative stress and curcumin-treated groups. The correlation coefficient ranged from 1.0 (indicating a strong positive correlation) to −1.0 (indicating a strong negative correlation), with 0 representing no correlation. Gentisic acid exhibited a strong negative association with Cys-Ala (R = −0.95, *p* < 0.01), and strong positive correlations with Chamanetin (R = 0.92, *p* < 0.01) and Glyoxal (R = 0.80, *p* < 0.01) (Figure 4A). A strong positive correlation was also observed between 3-hydroxybutyric acid and Oxodecanoylcarnitine (R = 0.94, *p* < 0.01), as well as between L-2-amino-3-oxybutyric acid and L-homoserine lactone (R = 0.83, *p* < 0.01). Glucosyl acid showed a strong negative correlation with L-homoserine lactone (R = −0.95, *p* < 0.01), as well as strong positive correlations with Oxodecanoylcarnitine (R = 0.81, *p* < 0.01) and 3-hydroxybutyric acid (R = 0.85, *p* < 0.01) (Figure 4B).

### 3.5. The Significance of Distinct Metabolites Within Metabolic Pathways

**Figure 5:**
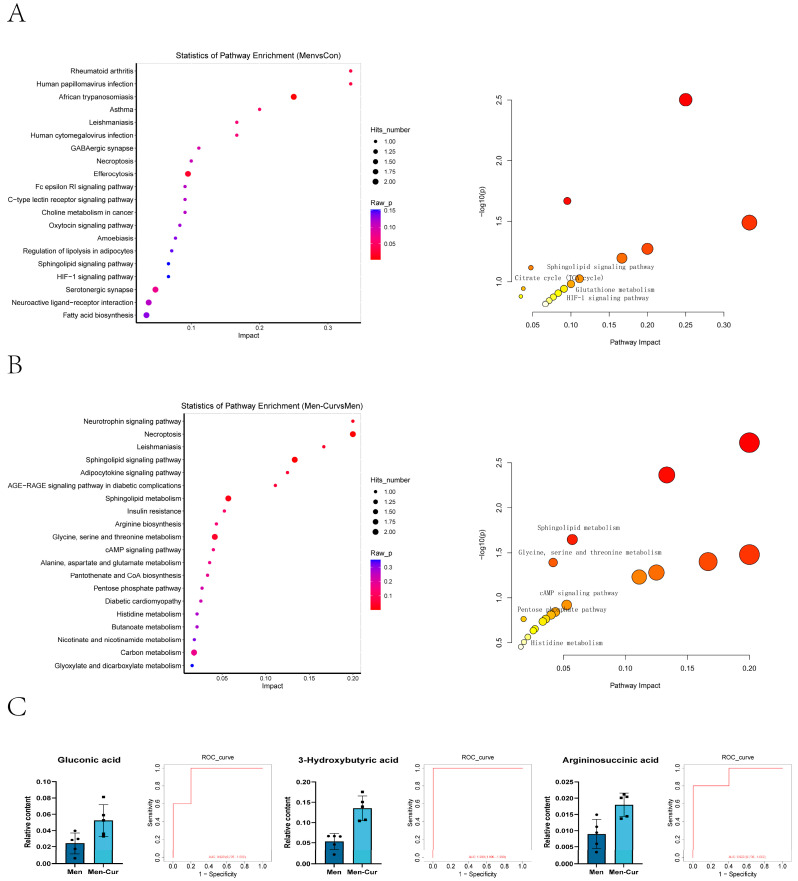
Enrichment map of metabolic pathways. (**A**) KEGG pathway analysis and abundance score of different metabolic pathways in oxidative stress group. (**B**) KEGG pathway analysis and abundance scores of different metabolic pathways in curcumin group. (**C**) Relative content of differential metabolites and ROC analysis in the curcumin treatment group. Note: (1) Con: citrate-Tris buffer + 0.1% DMSO; (2) Men: 0.1 mM menadione; (3) Men-Cur: 0.1 mM menadione + 25 μmol/L curcumin (*n* = 5 biological replicates).

Metabolic pathway analysis was conducted using the KEGG database to assess the involvement of differential metabolites in specific biochemical pathways. Several differential metabolites were enriched in pathways such as the citric acid cycle (TCA cycle), pentose and gluconate interconversion, arachidonic acid metabolism, vitamin B6 metabolism, the HIF-1 signaling pathway, and the cAMP signaling pathway, which are potentially important in the context of oxidative stress (Figure 5A). In the curcumin-treated group, pathways such as histidine metabolism, the pentose phosphate pathway, glycine/serine/threonine metabolism, sphingolipid metabolism, glyoxylate and dicarboxylate metabolism, and the cAMP signaling pathway were implicated in curcumin-mediated metabolic regulation (Figure 5B). Distinct metabolites were identified within these pathways, with notable examples including gluconic acid, 3-hydroxybutyric acid, and argininosuccinic acid. Relative content analysis and ROC curve evaluation were performed for these three metabolites. All exhibited AUC values greater than 0.8, suggesting strong predictive value for distinguishing semen status following curcumin treatment under oxidative stress (Figure 5C).

### 3.6. Multivariate Analysis of Lipid Profiles

**Figure 6:**
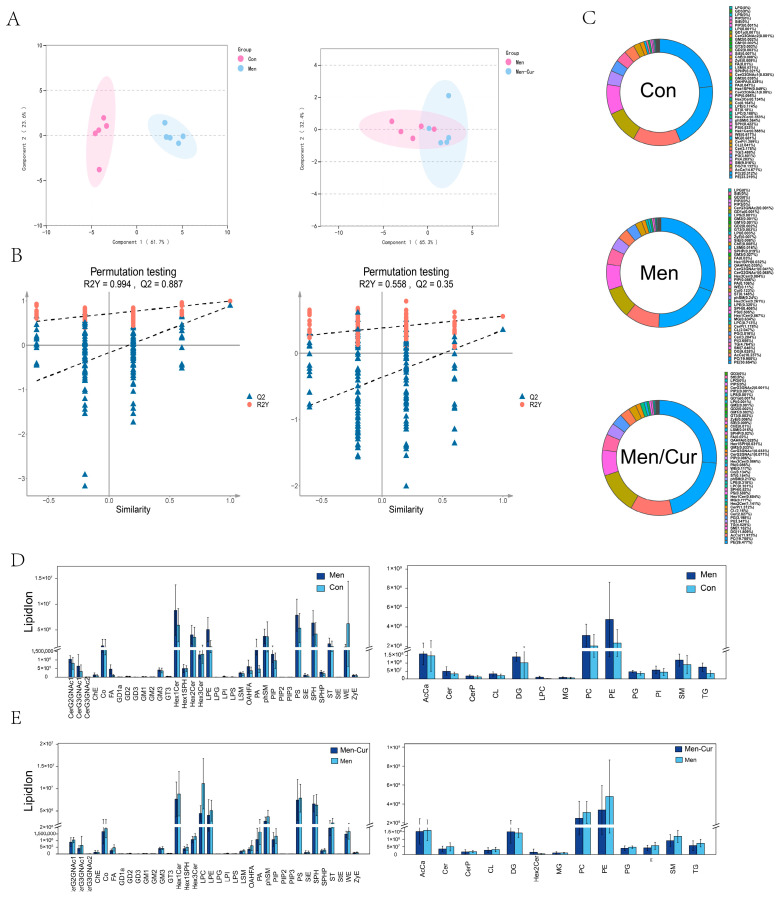
Lipidomics multivariate statistical analysis. (**A**) OPLS-DA score map is shown, with the abscissa representing the sample’s score on the first principal component and the ordinate representing the sample’s score on the second principal component. (**B**) Response ranking test map displays the correlation between randomly grouped Y and original group Y on the abscissa, while R^2^ and Q^2^ scores are represented on the ordinate. When R^2^ data exceeds Q^2^ data and the intercept between Q^2^ regression line and *Y*-axis is less than 0, it indicates that the model accurately describes the sample. (**C**) Statistical plot of the lipid classification in each group. (**D**) Statistics of lipid classification in oxitadive stress group. (**E**) Relative content of lipid classification in the curcumin-treated group. Note: 1. Con: citrate-Tris buffer + 0.1% DMSO; Men: 0.1 mM menadione; Men-Cur: 0.1 mM menadione + 25 μmol/L curcumin (*n* = 5 biological replicates).

To distinguish lipidomic differences between groups, an OPLS-DA model was applied. The results showed that the oxidative stress and curcumin-treated groups were distributed on opposite sides of the central axis, indicating clear separation between lipid profiles (Figure 6A). The model’s reliability and predictive power were supported by high R^2^Y and Q^2^ values. Permutation testing confirmed the absence of overfitting, as indicated by the negative intercept of the Q^2^ regression line on the *Y*-axis (Figure 6B). Major lipid classes were identified and quantified, with phosphatidylcholine (PC) and phosphatidylethanolamine (PE) showing higher relative abundance in both the oxidative stress and curcumin-treated groups, followed by acylcarnitine (AcCa) (Figure 6C). Additionally, the overall lipid composition was quantified in each group to assess curcumin’s influence on lipid regulation (Figure 6D,E).

### 3.7. Screening of Differential Lipid Molecules

**Figure 7:**
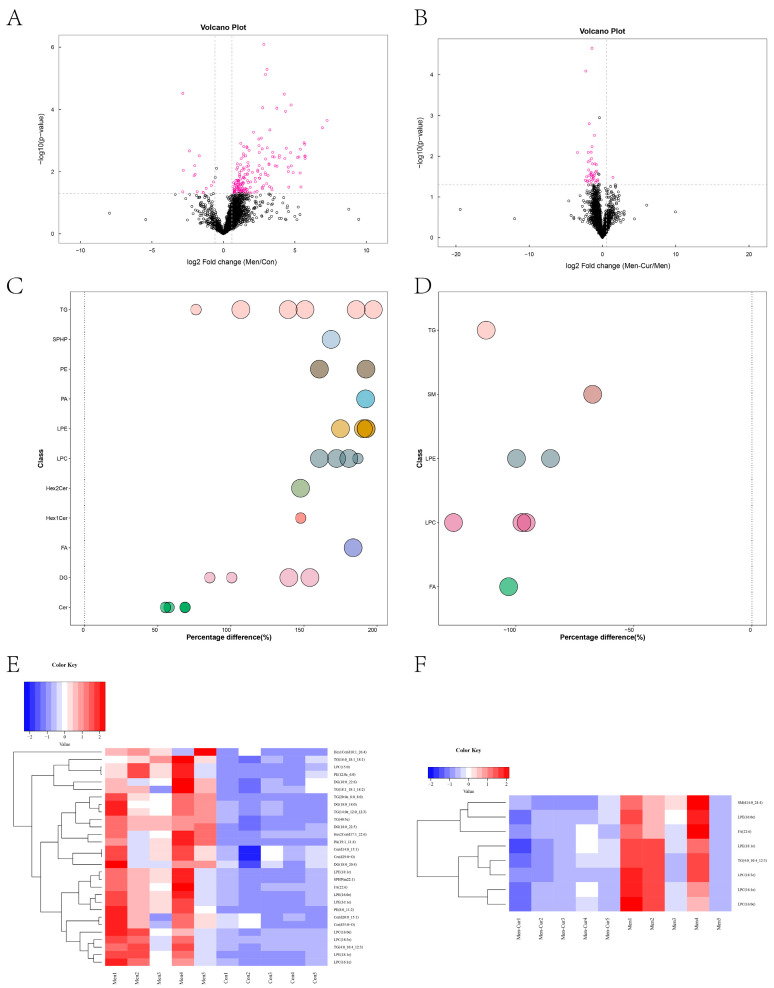
Molecular lipid screening and statistical analysis of differential lipids. (**A**,**B**) Volcanic plot of differential lipids. (**C**,**D**) Bubble maps of diverse lipids. Bubbles indicate significant difference lipid molecules; ordinate indicates each lipid subclass, distinguished by different colors; bubble size represents the significance of the difference. (**E**,**F**) Heat map of cluster analysis for differential lipid analysis. The left side shows the cluster, the row shows the differential lipid molecule, the right side shows the name, and the column shows the sample name. The blue part represents downregulation of expression. The red part represents upregulation. Note: (1) Con: citrate-Tris buffer + 0.1% DMSO; (2) Men: 0.1 mM menadione; (3) Men-Cur: 0.1 mM menadione + 25 μmol/L curcumin (*n* = 5 biological replicates).

Differential lipid analysis was conducted based on criteria of fold change (FC > 1.5 or FC < 0.67), VIP > 1, and *p* < 0.05. The screened lipids are listed in Table 2 and Table 3. Volcano plots (Figure 7A,B) highlighted lipid molecules that met the selection criteria. Under oxidative stress, key differential lipids included triglycerides (TG), lysophosphatidylcholine (LPC), and lysophosphatidylethanolamine (LPE) (Figure 7C). In contrast, curcumin treatment primarily altered fatty acids (FA) and LPCs (Figure 7D). Oxidative stress led to increased lipid accumulation (Figure 7E), whereas curcumin treatment significantly reduced lipid content (Figure 7F).

### 3.8. Correlation Between Different Lipids

**Figure 8:**
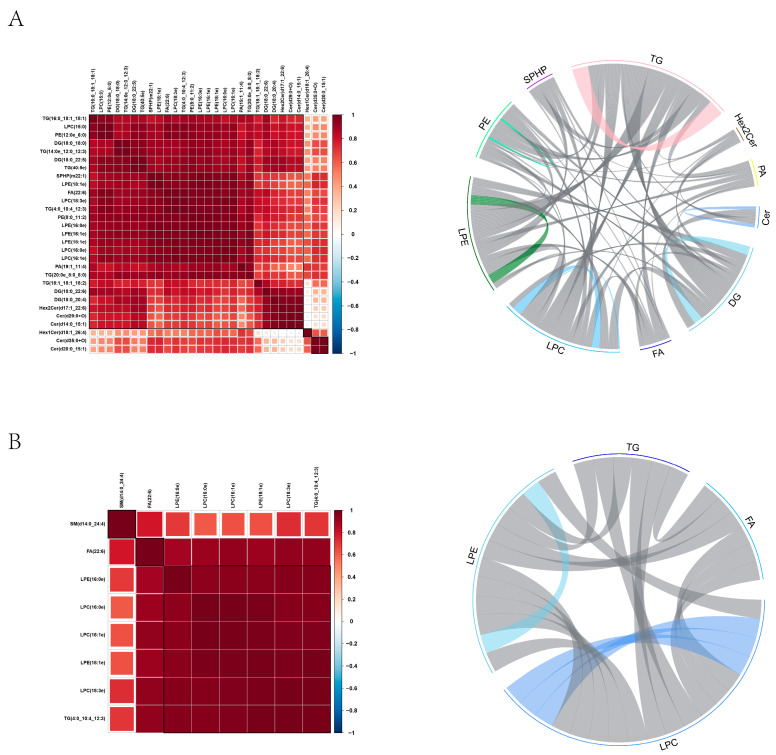
Correlation among differentially abundant lipids. (**A**) Pairwise correlation heatmap for the heat-stress group. (**B**) Pairwise correlation heatmap for the curcumin—treated group. Red denotes positive correlation and blue denotes negative correlation, with color saturation proportional to |r|. Lipids are hierarchically clustered to highlight co-regulation patterns.

To evaluate coordinated lipid responses, Pearson correlation analysis was performed. In the oxidative stress group, TG levels were positively correlated with diacylglycerol (DG), LPE, and LPC (Figure 8A), suggesting a shared lipid regulatory pattern under stress conditions. In the curcumin-treated group, LPC was positively correlated with LPE, FA, and TG, indicating lipid co-regulation during curcumin-mediated recovery (Figure 8B).

### 3.9. Effect of Curcumin on Sperm Ultrastructure Under Oxidative Stress

**Figure 9:**
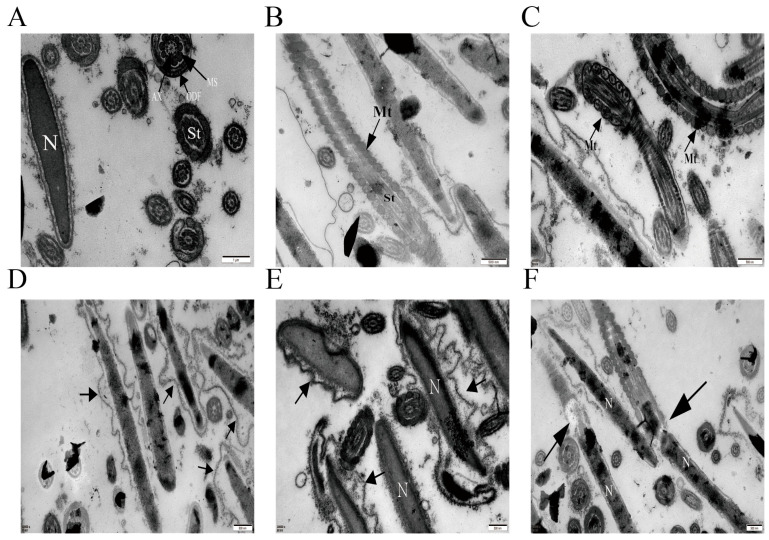
Transmission electron micrographs of spermatozoa following curcumin treatment (**A**–**C**) and under oxidative stress (**D**–**F**). (**A**) Representative near-normal ultrastructure in Cur-treated sperm. (**B**,**C**) Intact mitochondrial architecture with well-preserved cristae and a continuous mitochondrial sheath. (**D**,**E**) Plasma-membrane blebbing in oxidatively stressed sperm. (**F**) Mitochondrial swelling, vacuolization, and deformation under oxidative stress. Notes: The arrows point to the descriptions of the various structures they represent. Labels: N, nucleus; St, sperm tail; Mt, mitochondrion; AX, axoneme; MS, mitochondrial sheath; ODF, outer dense fibers.

**Figure 10:**
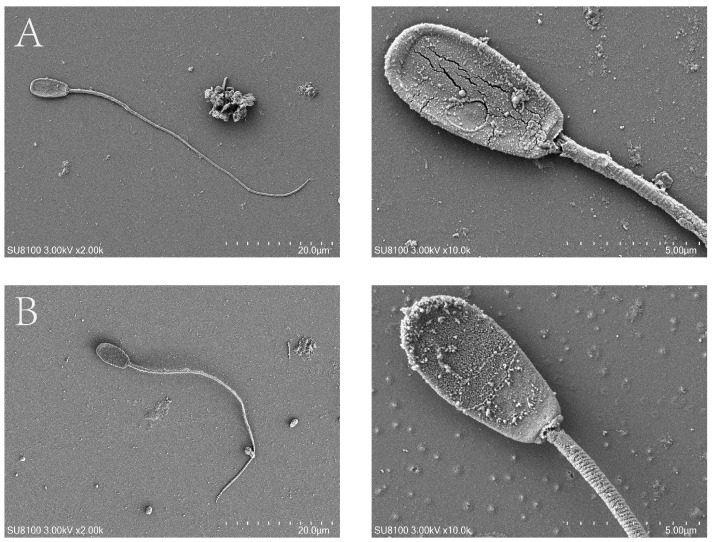
Scanning electron micrographs (SEM) of Hainan Black goat spermatozoa. (**A**) Surface morphology of sperm under oxidative stress. (**B**) Surface morphology of sperm after Cur treatment.

Transmission electron microscopy (TEM) revealed that curcumin preserved the ultrastructure of spermatozoa. Treated sperm exhibited intact plasma membranes closely apposed to the nuclear membrane, homogeneous chromatin distribution, and complete acrosomes with continuous membranes (Figure 9A). Mitochondria appeared structurally intact, with distinct inner and outer membranes and well-defined intermembrane spaces (Figure 9B,C). In contrast, oxidative stress induced apoptotic features including plasma membrane blebbing (Figure 9D,E), mitochondrial swelling, vacuolation, deformation, and loss of cristae (Figure 9F). Scanning electron microscopy (SEM) showed oxidative stress-related head deformities such as acrosomal disruption, flattening, or rupture (Figure 10A), which were significantly ameliorated by curcumin (Figure 10B).

### 3.10. Curcumin Alleviates Sperm Apoptosis Under Oxidative Stress

**Figure 11:**
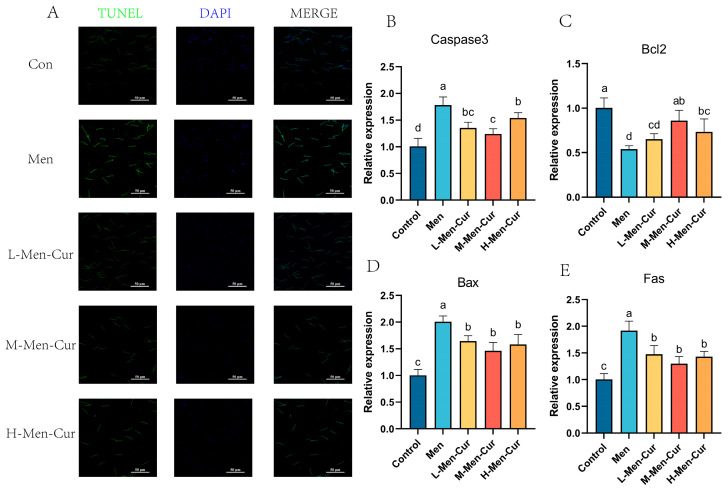
Effects of curcumin on sperm apoptosis. (**A**) TUNEL staining of spermatozoa and quantification of the apoptotic rate. (**B**–**E**) Relative mRNA abundance of apoptosis-related genes in spermatozoa: *Caspase-3*, *BCL2*, *BAX*, and *FAS*, respectively. Notes: 1. Con: citrate-Tris buffer + 0.1% DMSO; Men: 0.1 mM menadione; L-Men-Cur: 0.1 mM menadione + 5 μmol/L curcumin; M-Men-Cur: 0.1 mM menadione + 25 μmol/L curcumin; H-Men-Cur: 0.1 mM menadione + 50 μmol/L curcumin (*n* = 5 biological replicates). 2. Data are presented as mean ± SD. Groups not sharing a letter differ significantly (*p* < 0.05).

TUNEL staining demonstrated that curcumin significantly reduced sperm apoptosis rates compared to the oxidative stress group (Figure 11A). qPCR analysis showed that expression levels of pro-apoptotic genes (Fas, Bax, Caspase3) were significantly decreased, while anti-apoptotic Bcl2 expression was upregulated following curcumin treatment (Figure 11B–E). These findings indicate that curcumin mitigates oxidative stress-induced apoptosis in Hainan Black goat sperm, with the most pronounced effect observed at 25 μmol/L (M-Men-Cur).

## 4. Discussion

This study demonstrates that curcumin exerts significant protective effects against oxidative stress-induced damage in Hainan Black goat sperm. Through enhancement of antioxidant defense systems, curcumin improved semen quality, reduced apoptosis, and preserved sperm ultrastructure. Metabolomic analyses revealed that curcumin modulates key metabolic pathways related to energy production and oxidative defense, with gluconic acid, 3-hydroxybutyric acid, and argininosuccinic acid identified as central metabolites.

### 4.1. Effects of Curcumin on Sperm Metabolism of Hainan Black Sheep Under Oxidative Stress

Spermatozoa primarily rely on glucose metabolism via the pentose phosphate pathway (PPP) to generate NADPH and maintain redox homeostasis [27]. Elevated gluconic acid levels, a byproduct of glucose oxidation, have been implicated in promoting oxidative stress [28]. The elevation of gluconic acid following curcumin treatment suggests activation of the PPP to sustain ATP levels and sperm function [29]. Concurrently, NADPH derived from the PPP serves as a cofactor for regenerating reduced glutathione (GSH) from its oxidized form (GSSG), bolstering antioxidant defenses against oxidative damage [30,31]. Furthermore, 3-hydroxybutyric acid, elevated in curcumin-treated groups, enhances mitochondrial membrane potential, reduces DNA fragmentation, and improves mitochondrial resilience against oxidative stress [32,33]. Curcumin also restored argininosuccinic acid levels—a metabolite depleted in malformed sperm—surpassing concentrations observed in the oxidative stress group [34]. Collectively, these metabolic shifts underscore curcumin’s role in rebalancing energy metabolism, antioxidant activity, and structural integrity in stressed sperm.

### 4.2. Effects of Curcumin on Sperm Lipids of Hainan Black Sheep Under Oxidative Stress

Lipidomic analyses revealed pronounced remodeling of sperm lipid profiles following curcumin treatment. Oxidative stress induced a marked accumulation of lysophosphatidylcholines (e.g., LPC(16:0e), LPC(16:1e), LPC(18:3e)), a signature of membrane disruption linked to impaired sperm integrity [35]. Curcumin attenuated this LPC enrichment, suggesting preservation of membrane architecture. Triglycerides (TGs), important energy stores for spermatogenesis [36], were elevated under oxidative stress. Although physiological TG levels can correlate positively with motility in certain species (e.g., white salmon) [37], pathological TG accumulation—reported here and in diabetic rodent models—perturbs membrane dynamics and compromises motility [38]. Consistently, Slc22a14-deficient mice exhibit TG-driven metabolic dysfunction with reduced β-oxidation and depleted tricarboxylic-acid (TCA) intermediates [39]. Curcumin normalized TG levels, likely mitigating these adverse effects.

Sperm membranes have an unusual lipid composition with a high contribution of sphingolipids [40]. In rat sperm heads, sphingomyelin (SM) can be converted to ceramide during the acrosome reaction, facilitating membrane reorganization [41]. However, higher SM content has been observed in stallion sperm with poor freezing resistance and correlates negatively with motility and membrane integrity [42]. Curcumin reduced SM abundance, a change potentially stabilizing the membrane and limiting stress-induced remodeling. Overall, curcumin counters oxidative injury by restoring lipid homeostasis: lowering peroxidation-prone LPCs, normalizing energy-critical TGs, and modulating SM-dependent membrane dynamics.

### 4.3. Effect of Curcumin on Oxidative Stress-Induced Sperm Apoptosis of Hainan Black Goat

Apoptosis is a major determinant of sperm quality, affecting motility, membrane integrity, and acrosomal function [43,44]. Notably, cryo-induced apoptosis negatively correlates with motility and plasma membrane integrity during sperm preservation [45,46]. In this study, apoptosis in goat sperm exposed to oxidative stress—with or without curcumin—was evaluated by TUNEL in combination with DAPI staining, a well-established approach in andrology research [47]. Curcumin markedly reduced the proportion of apoptotic sperm, underscoring apoptosis as a sensitive indicator of oxidative damage in goat semen.

Ultrastructural analyses revealed canonical features of apoptosis, including membrane blebbing, apoptotic body formation, nuclear envelope defects, and nuclear fragmentation, mirroring observations in human sperm [48]. Particularly prominent were mitochondrial alterations, consistent with mitochondria-dependent apoptotic signaling [49,50]. Oxidative stress elevated endogenous ROS and malondialdehyde (MDA) while lowering the activities of SOD, CAT, and GSH-Px, indicating both excessive ROS generation and diminished antioxidant performance. Elevated ROS drives lipid peroxidation, compromises membrane quality, and precipitates loss of mitochondrial membrane potential (MMP), cytochrome-c release, and caspase activation—hallmarks of mitochondria-mediated apoptosis [51,52,53,54,55,56]. In contrast, curcumin, acting as an effective ROS scavenger, curtailed ROS accumulation, improved antioxidant enzyme status, restored MMP, and ultimately restrained apoptosis. Collectively, these data support a model in which oxidative stress triggers ROS-driven mitochondrial damage and mitochondria-dependent apoptosis in goat spermatozoa, while curcumin interrupts this cascade by re-establishing redox balance and mitochondrial function.

## 5. Conclusions

Curcumin (25 μmol/L) exhibits significant protective effects against oxidative stress-induced damage in Hainan Black goat sperm. It enhances antioxidant capacity, preserves mitochondrial function, restores membrane integrity, and reduces apoptosis. Integrated metabolomic and lipidomic analyses revealed that curcumin modulates critical pathways involved in energy metabolism, redox balance, and lipid homeostasis, with gluconic acid, 3-hydroxybutyric acid, and argininosuccinic acid identified as key biomarkers. These findings provide mechanistic insights into curcumin’s role in mitigating oxidative damage and highlight its potential as a natural antioxidant for improving semen quality and fertility in livestock breeding programs.

## Figures and Tables

**Table 1:** Primer sequence information to study of Hainan Black Goat spermatozoa.

Gene Name	Primer Sequences (5′to3′)	Accession Number
*β-actin*	F: GCGGCATTCACGAAACTACC	NM_001314342.1
	S: ACTCCTGCTTGCTGATCCAC	
*BAX*	F: CGAGTGTCTGAAGCGCATTG	XM_013971446.2
	S: GGGCCTTGAGCACCAGTTTG	
*BCL-2*	F: AGGCTCACAGCACACTCTTC	XM_018039337.1
	S: GGCCTGTGGGCTTCACTTAT	
*FAS*	F: GCACACAATATGGACCCCCA	NM_001285629.1
	S: CATGCTGTAGCCTACGAGGG	
*CASPASE-3*	F: CAGACCTGGACTGTGGTATTGAG	NM_001286089.1
	S: AGCAGAATCGGTGGAAAAGGAGC	

**Table 2:** Differentially abundant lipids between the Men group and Control groups. Notes: 1. Con: citrate-Tris buffer + 0.1% DMSO; Men: 0.1 mM menadione; 2. FC: fold-change; VIP: variable importance in projection.

Lipid Ion	Lipid Group	Ion Formula	FC	*p*-Value	VIP
PE(18:1_22:6)-H	PE(40:7)-H	C45 H75 O8 N1 P1	0.280	0.032	1.043
Cer(d46:5)+HCOO	Cer(d46:5)+HCOO	C47 H84 O5 N1	9.238	0.032	3.214
Cer(m38:0+O)+HCOO	Cer(m38:0+O)+HCOO	C39 H78 O5 N1	0.152	0.039	1.052
CerP(d49:3)-H	CerP(d49:3)-H	C49 H93 O6 N1 P1	0.205	0.022	1.744
Hex1Cer(d16:1_20:0)-H	Hex1Cer(d36:1)-H	C42 H80 O8 N1	0.412	0.045	1.131
Hex2Cer(d30:1)+HCOO	Hex2Cer(d30:1)+HCOO	C43 H80 O15 N1	0.403	0.048	1.572
Hex2Cer(d35:5)-H	Hex2Cer(d35:5)-H	C47 H80 O13 N1	0.149	0.039	1.269
Hex2Cer(d16:0_20:4)+HCOO	Hex2Cer(d36:4)+HCOO	C49 H86 O15 N1	0.473	0.049	1.235
LPC(16:0e)+HCOO	LPC(16:0e)+HCOO	C25 H53 O8 N1 P1	4.006	0.021	1.759
LPC(16:1e)+HCOO	LPC(16:1e)+HCOO	C25 H51 O8 N1 P1	4.483	0.020	3.325
LPC(22:6)+HCOO	LPC(22:6)+HCOO	C31 H51 O9 N1 P1	0.464	0.023	1.580
LPE(16:1e)-H	LPE(16:1e)-H	C21 H43 O6 N1 P1	6.580	0.010	2.629
OAHFA(57:10)-H	OAHFA(57:10)-H	C57 H91 O4	0.360	0.040	1.166
AcCa(16:0)+H	AcCa(16:0)+H	C23 H46 O4 N1	0.539	0.033	13.144
LPC(18:3e)+H	LPC(18:3e)+H	C26 H51 O6 N1 P1	6.107	0.020	2.372
PC(40:10)+H	PC(40:10)+H	C48 H77 O8 N1 P1	0.345	0.049	2.214
TG(4:0_10:4_12:3)+NH4	TG(26:7)+NH4	C29 H44 O6 N1	5.391	0.020	4.430
AcCa(14:0)+H	AcCa(14:0)+H	C21 H42 O4 N1	0.394	0.010	11.315
PE(18:1_22:6)-H	PE(40:7)-H	C45 H75 O8 N1 P1	0.280	0.032	1.043

**Table 3:** Differentially abundant lipids between the Curcumin treatment group and Men group. Notes: 1. Curcumin treatment group: 0.1 mM menadione + 25 μmol/L curcumin; Men: 0.1 mM menadione; 2. FC: fold-change; VIP: variable importance in projection.

Lipid Ion	Lipid Group	Ion Formula	FC	*p*-Value	VIP
FA(22:6)-H	FA(22:6)-H	O2 H31 C22	0.320	0.050	1.532
LPC(16:0e)+HCOO	LPC(16:0e)+HCOO	C25 H53 O8 N1 P1	0.345	0.041	3.022
LPC(16:1e)+HCOO	LPC(16:1e)+HCOO	C25 H51 O8 N1 P1	0.353	0.034	5.384
LPE(16:0e)-H	LPE(16:0e)-H	C21 H45 O6 N1 P1	0.334	0.029	1.215
LPC(18:3e)+H	LPC(18:3e)+H	C26 H51 O6 N1 P1	0.225	0.032	5.940
LPE(18:1e)+H	LPE(18:1e)+H	C23 H49 O6 N1 P1	0.402	0.038	1.101
SM(d14:0_24:4)+H	SM(d38:4)+H	C43 H82 O6 N2 P1	0.496	0.015	4.116
TG(4:0_10:4_12:3)+NH4	TG(26:7)+NH4	C29 H44 O6 N1	0.280	0.030	9.385

## Data Availability

Data are contained within the article.

## Abbreviations

ROS	Reactive oxygen species
PUFA	Polyunsaturated fatty acids
Cur	Curcumin
MDA	Malondialdehyde
SOD	Superoxide dismutase
CAT	Catalase
T-AOC	Total antioxidant capacity
ATP	Adenosine triphosphate
MMP	Mitochondrial membrane potential.
FC	Fold change
LC-MS	Liquid Chromatograph Mass Spectrometer
PCA	Principal component analysis
OPLS-DA	Orthogonal partial least squares-discriminant analysis
VIP	Variable importance projection
DAMs	Differential accumulation metabolites
KEGG	Kyoto Encyclopedia of Genes and Genomes
TG	Triglycerides
DG	Diglycerides
PE	Phosphatidylethanolamine
AcCa	Acylcarnitine
TUNEL	Terminal deoxynucleotidyl transferase-mediated nick end labeling
SEM	Scanning Electron Microscopy
TEM	Transmission Electron Microscopy
CerP	Phosphate ceramide
chE	Cholesterol lipid
CL	Cardiolipin
Co	Coenzyme
DG	Glycerodiamide
FA	Fatty acid
GD	Ganglioside
GM3	Ganglioside
Hex1Cer	Hexanceramide
SPH	Sphingomyelin
LPC	Lysophosphatidyl choline
LPS	Lytic ophosphatidylserine
LMG	Monoacylglycerol
OAHFA	O-acyl -1-hydroxy fatty acid
PA	Phosphatidic acid
PC	Phosphatidylcholine:
PG	Phosphatidylglycerol
PhSM	Phytosphingosine
PIP	Phosphatidyllinositol-4-phosphoric acid
PS	Phosphatidylserine:
SiE	Sitotenyl ester
PI	Phosphatidylinositol
SM	Sphingomyelin phospholipid
ST	Sterol lipid
StE	Stigmatyl ester
SH	Sphingosine
SPHP	Sphingosine phosphate
